# Publisher Correction: Motif-based substrate mapping of the receptor-like cytoplasmic kinase BIK1 reveals novel components and regulatory nodes of plant immunity

**DOI:** 10.1038/s41477-026-02255-2

**Published:** 2026-03-03

**Authors:** Ryan Toth, Sera Choi, Marie Le Naour--Vernet, Florian Schwanke, Jared L. Johnson, Estee E. Tee, Tomer M. Yaron-Barir, Eleanor Khochaba, Paul Derbyshire, Anka Colo, Philipp Köster, Emily M. Huntsman, Laura Herold, Yoonyoung Lee, Álvaro D. Fernández-Fernández, Hee-Kyung Ahn, Julian Dindas, Marta Bjornson, Jack Rhodes, Beibei Song, Weibing Wang, Marija Smokvarska, Emmanuelle M. Bayer, Jian-Min Zhou, Lewis C. Cantley, Jonathan D. G. Jones, Kyle W. Bender, Frank L. H. Menke, Christine Faulkner, Cyril Zipfel, Thomas A. DeFalco

**Affiliations:** 1 Institute of Plant and Microbial Biology, Zürich-Basel Plant Science Center, University of Zürich, Zürich, Switzerland; 2 Meyer Cancer Center, Weill Cornell Medicine, New York, NY USA; 3 Department of Cell Biology, Harvard Medical School, Boston, MA USA; 4 Dana-Farber Cancer Institute, Harvard Medical School, Boston, MA USA; 5 Cell and Developmental Biology, John Innes Centre, Norwich, UK; 6 Department of Pediatrics, Boston Children’s Hospital, Harvard Medical School, Boston, MA USA; 7 Department of Biology, Western University, London, Ontario Canada; 8 The Sainsbury Laboratory, University of East Anglia, Norwich Research Park, Norwich, UK; 9 Yazhouwan National Laboratory, Sanya, China; 10 Institute of Genetics and Developmental Biology, Chinese Academy of Sciences, Beijing, China; 11 Laboratoire de Biogenèse Membranaire, UMR5200, CNRS, Université de Bordeaux, Villenave d’Ornon, France; 12 Present Address: Plant Breeding Institute, School of Life and Environmental Sciences, University of Sydney, Cobbitty, New South Wales Australia; 13 Present Address: Institute of Plant Biology and Biotechnology, University of Münster, Münster, Germany; 14 Present Address: Englander Institute for Precision Medicine, Institute for Computational Biomedicine, Weill Cornell Medicine, New York, NY USA; 15 Present Address: Institute of Molecular Plant Sciences, University of Edinburgh, Edinburgh, UK; 16 Present Address: Department of Plant Sciences, University of California, Davis, CA USA; 17 Present Address: School of Biological Sciences, Nanyang Technological University, Singapore, Singapore

**Keywords:** Pattern recognition receptors in plants, Plant signalling

Correction to: *Nature Plants* 10.1038/s41477-025-02218-z, published online 9 February 2026.

In the version of the article initially published, the colours were missing from the keys in Figs. 2d, 2f and 3i and have now been added to the HTML and PDF versions of the article. Additionally, Paul Derbyshire was originally listed with affiliation 7 but this has now been corrected to affiliation 8 (The Sainsbury Laboratory, University of East Anglia, Norwich Research Park, Norwich, UK).

